# Editorials

**Published:** 1892-01

**Authors:** 


### HOMŒOPATHIC PHYSICIAN,

A MONTHLY JOURNAL OF

HOMŒOPATHIC MATERIA MEDICA AND CLINICAL MEDICINE.

"If our school ever gives up the strict inductive method of Hahnemann, we are lost, and deserve only to be mentioned as a caricature in the history of medicine."—CONSTANTINE HERING.

Vol. XII. JANUARY, 1892. No. 1.

### EDITORIAL.

SALUTATORY.—In entering upon a new year it seems desirable to once more call the attention of the readers of this journal to the object for which the journal was founded.

As has been stated in the December number, at the time it was started there was no journal that could be depended upon to teach the pure doctrine, and to maintain it against all foes. There was a large number of so-called homœopathic journals before the profession. But their principal objects were to extend their circulation, and to catch advertisements. To do this they sought only how to cater to what may be called the public opinion of the profession. They had no idea of educating it. They therefore were servile followers of that public opinion, notwithstanding its want of enlightenment. They did not dare to be leaders of it lest it would impair their circulation.

Most of them are still of this character, there being only two or three honorable exceptions. Therefore this journal finds itself still with a mission, that mission being to teach Homœopathy as taught by the immortal Hahnemann.

Whether the pursuing of that mission shall increase or decrease its circulation, whether it shall make it profitable in a commercial sense, or whether it shall reduce it to bankruptcy; whether it shall meet the approval of the other journals in the profession or secure their condemnation; whether it shall raise a host of friends or a cloud of enemies; whether it shall receive reward or persecution, that mission will be faithfully pursued in the future exactly as in the past.

The three great principles of Homœopathy are the similar remedy, the single drug, the minimum dose.

The only method possible to conquer a sick condition is the similar remedy. To select the similar remedy we must have provings. The proving shows the sphere of action of the drug, its scope. When the drug, that by testing upon a healthy person, gives certain characteristic indications, is applied to a sick condition in which those characteristics crop out spontaneously, then we are assured that we have brought about a collision or conflict between the spontaneous disease influences upon the one hand, and of the artificial drug power upon the other. It now remains only to so graduate the dose that the drug power shall be just sufficient to overcome the disease influence, and not substitute any damage of its own. Here is the guiding star of the homœopathist who wishes to really *cure* disease without injuring his patient by drug action. This would seem to be so rational and obvious a principle that it would be eagerly embraced and practiced by all earnest medical men, and more especially by such as have braved odium sufficiently to avow themselves homœopathists. Yet such is not the case. It is neglected, it is rejected, it is even unknown. Therefore, it becomes the duty of this journal to present it again and again; to illustrate it in every possible way until it has become so familiar that all shall acknowledge its truth and become its enthusiastic apostles. The mission of THE HOMŒOPATHIC PHYSICIAN, therefore, is to *teach* the profession a great principle, and every contributor who adds a single page of wise observation, discussion, or clinical experience by which some brother in the craft is benefited becomes a teacher, makes himself a part of the purpose of the journal, identifies himself with the journal, increases its influence, and helps to make it the leader of the opinion of the profession.

It is now the pleasant duty of the editor to thank the many readers of this journal for their loyal support and indorsement of the journal during the past year. Not alone by their subscriptions have they ensured its prosperity, but by their contributions to its pages. A large number of these contributions is still unpublished, so that we begin the new year with quite a rich store of instructive articles.

---

NOT FOR SALE.—Reports having reached the editor that this journal is for sale, or is about to cease publication, I take this opportunity to state most emphatically that THE HOMŒOPATHIC PHYSICIAN is not for sale or for barter under any conditions with any person or society; that it will continue as heretofore to be published without any change whatever.

WALTER M. JAMES,
*Editor and Publisher.*

